# Multifunctional Inorganic Nanoparticles: Recent Progress in Thermal Therapy and Imaging

**DOI:** 10.3390/nano6040076

**Published:** 2016-04-18

**Authors:** Kondareddy Cherukula, Kamali Manickavasagam Lekshmi, Saji Uthaman, Kihyun Cho, Chong-Su Cho, In-Kyu Park

**Affiliations:** 1 Department of Biomedical Science and BK21 PLUS Centre for Creative Biomedical Scientists, Chonnam National University Medical School, Gwangju 501-746, Korea; cherrikonda@gmail.com (K.C.); kamali.mvasagam@gmail.com (K.M.L.); sajiuthaman@gmail.com (S.U.); 2 Department of Agricultural Biotechnology and Research Institute for Agriculture and Life Sciences, Seoul National University, Seoul 151-921, Korea; andrew.kihyun.cho@gmail.com

**Keywords:** inorganic nanoparticles, surface plasmon resonance, alternate magnetic field, photothermal therapy, imaging, image-guided therapy

## Abstract

Nanotechnology has enabled the development of many alternative anti-cancer approaches, such as thermal therapies, which cause minimal damage to healthy cells. Current challenges in cancer treatment are the identification of the diseased area and its efficient treatment without generating many side effects. Image-guided therapies can be a useful tool to diagnose and treat the diseased tissue and they offer therapy and imaging using a single nanostructure. The present review mainly focuses on recent advances in the field of thermal therapy and imaging integrated with multifunctional inorganic nanoparticles. The main heating sources for heat-induced therapies are the surface plasmon resonance (SPR) in the near infrared region and alternating magnetic fields (AMFs). The different families of inorganic nanoparticles employed for SPR- and AMF-based thermal therapies and imaging are described. Furthermore, inorganic nanomaterials developed for multimodal therapies with different and multi-imaging modalities are presented in detail. Finally, relevant clinical perspectives and the future scope of inorganic nanoparticles in image-guided therapies are discussed.

## 1. Introduction

**Figure 1:**
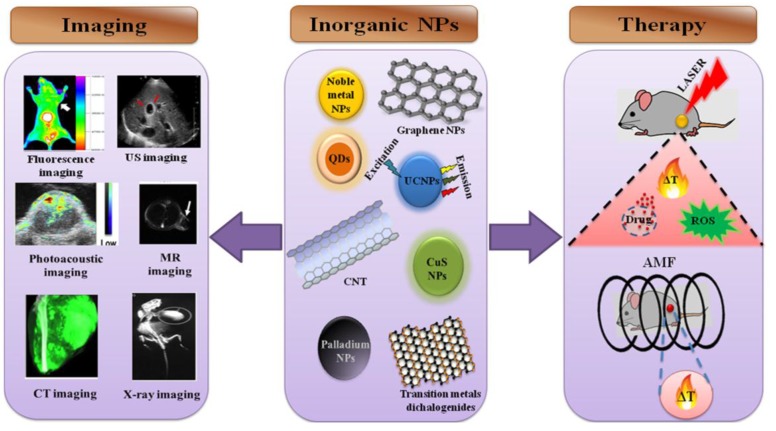
Scheme illustrating the potential of inorganic nanoparticles in heat-induced therapies and imaging. US: ultrasound; MR: magnetic resonance; CT: computed tomography; QD: quantum dot; UCNP: upconversion nanoparticles; CuS: copper sulfide; CNT: carbon nanotube; AMF: alternate magnetic field; ROS: reactive oxygen species.

Cancer treatment is mainly performed with chemotherapy, radiation, and surgery. However, all these strategies have limitations, such as toxic side effects, healthy cell damage, and tumor recurrence. Researchers have investigated alternative and complementary therapies to completely eliminate tumor cells and prevent cancer recurrence. In the past few decades, hyperthermia has been used to kill exclusively the tumor cells. Nanoparticles using organic molecules have been widely investigated for thermal therapy and imaging [1,2,3]. Although organic dye molecules with low tissue absorbance and enhanced photothermal effects have been used for thermal therapy, photobleaching remains one of their major drawbacks [4]. Recently, inorganic nanoparticles have attracted attention in the fields of heat-induced cancer therapy and imaging owing to their optical, magnetic and their inertness; thus, they provide an attractive alternative for image-guided therapies, as shown in Figure 1. Inorganic nanoparticles have exhibited diverse physical properties, such as fluorescence, near-infrared (NIR) absorption, and Raman enhancement and applications such as photoacoustic imaging (PAI) and magnetic resonance imaging (MRI) [5]. However, clearance of inorganic nanoparticles and their long term toxicity need to be examined very carefully before using in clinics. Surface modification of the nanoparticles by conjugating molecules such as polyethylene glycol (PEG) would change the circulation scenario of the nanoparticles *in vivo*, and is excreted from the body without eliciting any potential toxicity. Carbon materials such as graphene and carbon nanotubes, due to their nucleus penetration ability, exhibit genotoxicity. By modifying the surface with PEG, toxicity can be reduced and it is excreted from the mice gradually [6]. Thus, by carefully designing the formulation these nanoparticles, potential barriers such as intrinsic toxicity and clearance can be avoided for a better therapeutic outcome.

In the past few decades, photothermal therapy (PTT) has attracted increasing interest as an effective cancer treatment [7]. Large electric fields are induced at the surface level of metal nanoparticles by the coherent oscillation of electrons in the conduction band when they interact with resonant electromagnetic radiation. The rapid relaxation of these excited electrons can produce heat locally and can be utilized to kill cancer cells in thermal-based therapies. This electric field enhances the photo-physical properties of the nanoparticles and is termed the surface plasmon resonance (SPR) [8]. The surface properties of noble metals are greatly enhanced when their sizes are reduced to the nanoscale owing to their strong SPR. Metallic nanoparticles offer various advantages for PTT because they exhibit higher absorption cross-section compared to organic dyes and thereby reduce the energy required for laser treatment, enabling a minimally invasive therapy. In addition, metallic nanoparticles do not undergo photobleaching upon irradiation and thus show high photostability and achieve effective laser therapy [9]. Two mechanisms have been proposed to describe cell death caused by PTT: apoptosis and necrosis. Apoptosis is an active and controlled process that induces cell death without triggering immune and inflammatory reactions whereas necrosis is a passive process resulting in membrane damage [10] and thus leading to inflammation by releasing damage-associated molecular pattern molecules (DAMPs) [11].

Magnetic hyperthermia (MHT) has been used for cancer treatment as early as 1957. In this method, which has few side effects, tumor cells are supplied with heat using magnetic nanoparticles and an alternating magnetic field (AMF) [12,13,14,15]. Temperatures ranging between 42 and 46 °C can effectively kill the cancer cells while sparing the healthy ones during AMF application [16]. AMF heating has several advantages over other heating methods, such as tumor temperature regulation and deep penetration [17]. Recently, carbon-based nanomaterials, such as graphene and carbon nanotubes, have attracted attention in the research of heat-induced therapies such as PTT owing to their unusual absorption properties in the NIR region [18]. In reduced graphene oxide (rGO), NIR absorption is due to the creation of a large electron density by displacing the oxygen atoms [19]. Carbon nanomaterials have been proven to be efficient PTT agents owing to their high photon-to-thermal energy conversion efficiency and high absorption cross section in the NIR region [20].

Thermal therapies use either light or magnetism as source for heating the tumor cells. MHT has been studied in the humans for the treatment of glioblastoma and prostate cancer [21,22]. On the other hand, PTT will soon find its application in clinics owing to its promising results in animal models. However, both approaches are limited by certain factors such as dosage, toxicity, *etc.* Multimodal therapies help us achieve the enhanced therapeutic effect by overcoming the drawbacks of individual therapies. Thermal therapies are often integrated with the other conventional therapies such as chemotherapy, radiation therapy, *etc.* to enhance their therapeutic potential and achieve combinatorial anti-cancer effects [22,23,24]. Multimodal therapies have been demonstrated to be effective strategies for the complete elimination of tumor cells and have provided better therapeutic efficacy than single-mode therapies [23,24,25,26,27]. Multifunctional nanoparticles, which provide multimodal imaging, are essential for detecting and treating the cancer at very early stages. Inorganic nanoparticles have been engineered to offer multimodal imaging and to collect information from the tumor site, thus enabling the clinicians to treat cancer effectively. Several inorganic nanoparticles have been designed to be multifunctional theranostic agents and exhibit favorable properties for multimodal imaging [28,29,30].

## 2. Surface Plasmon Resonance-Based Thermal Therapy

Thermal ablation of plasmonic nanoparticles proved to be an effective strategy because of its unique properties of plasmonic nanoparticles such as deep penetration into human tissue with minimal damage and thus aids in a thermal therapy with biocompatibility and reduced toxicity to the healthy cells [31].

### 2.1. Nanoscale Gold Particles

Nanoscale gold particles (NGP) are the extensively studied plasmonic nanomaterial for thermal therapy because of their enhanced photostability, higher light-to-heat conversion efficiencies, improved biocompatibility and importantly plasmon resonance in the NIR region [32]. NGPs have much stronger light absorption and emission properties than any other organic dye molecules owing to their SPR properties; hence, they are very attractive option for PTT [33]. At present, three major classes of NGPs are extensively used for PTT: (1) gold nanorods (NRs); (2) gold nanoshells; and (3) gold nanocages. The photothermal properties of NGPs mainly depend on the size, shape, and dielectric constant of the medium. NGPs have strong absorbance in the UV region although the SPR absorption red-shifts to the NIR region after aggregation. Metallic nanoparticles such as gold nanoparticles tends to aggregate due to van der Waals forces and hydrophobic forces [34]. Spherical gold nanoparticles attained importance in thermal therapy due to its aggregation properties and high NIR absorption, but it suffers from low disintegration and low tissue clearance which eventually causes potential toxicity [35]. Gold NRs exhibit higher SPR absorption than spherical particles owing to their aspect ratio. The SPR red shift reaches a maximum with an increase in the aspect ratio of the gold NRs. Similarly, a reduction of the ratio of the thickness of gold nanoshells to their core diameter greatly enhances the SPR wavelength [36,37,38].

At present, different morphologies of gold nanomaterials are explored to achieve enhanced therapeutic outcome. One such strategy was to coat the gold nanoparticles with amorphous SiO_2_ to form the gold nanoaggregates. This coating of SiO_2_ on gold nanoparticles which is greater than 1.4 nm showed improved biocompatibility and also served as a dielectric spacer to tune the PTT [39]. PTT efficiency of nanoaggregates was comparable with the other morphologies such as gold NRs with similar Au concentrations (30 mg/L) [40]. Even though the hydrophilic property of silica is used for the biodistribution of nanomaterials, it also interacts with the normal tissues and causes subsequent damage [41]. Therefore, amphiphilic polymers were grafted on NGPs to form dense self assembled structures. PTT studies showed a Δ*T* of 23 °C and esterase dependent disintegration of nanoparticles and successful cellular damage *in vivo* [42].Polymers that induces thermo responsive properties were formulated with gold NRs as nanocomposites to facilitate the tissue penetration and reduced size for a better cytotoxic effects [43]. NGPs are employed in various imaging techniques, such as photoacoustic imaging (PAI), two-photon luminescence microscopy, and dark-field microscopy [44,45,46]. Colloidal gold nanoparticles have also been used as enhancers for X-ray computed tomography (CT) imaging owing to the high atomic number and high absorption coefficient of gold. Gold nanoparticles provide greater contrast and less interference compared to the conventionally used iodine [47]. They have also been observed to increase the contrast of magnetic resonance imaging (MRI) contrast agents, such as gadolinium and iron oxide nanoparticles, by enhancing their retention and optical properties [48].

**Figure 2:**
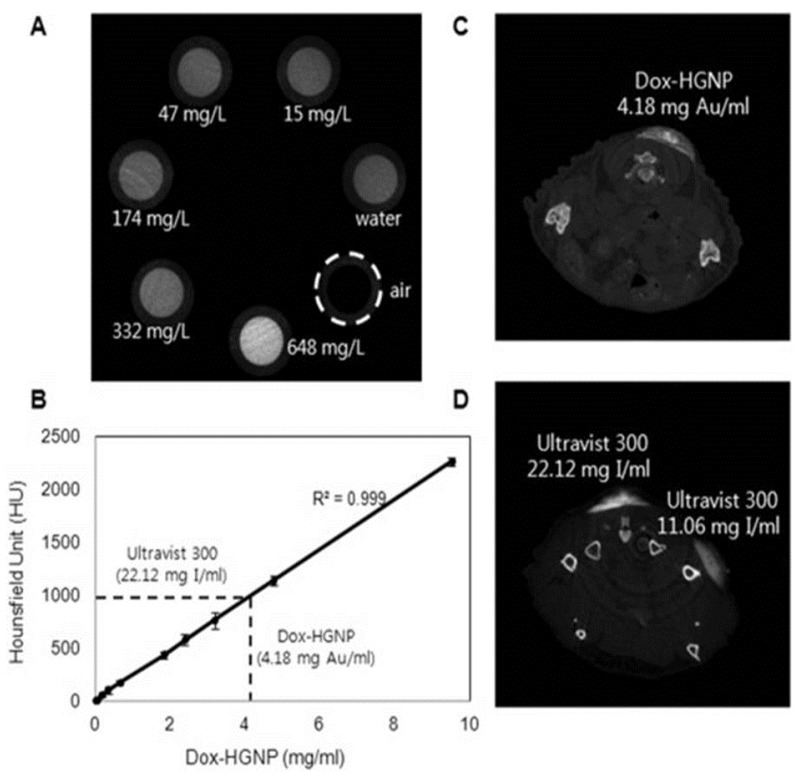
*In vitro* and *in vivo* micro-CT images: (**A**) concentration-dependent CT images of air, distilled water, and doxorubicin-loaded hollow gold nanoparticles (Dox-HGNPs); (**B**) X-ray absorption of Dox-HGNP and Ultravist 300; (**C**) cross-sectional CT image in the back skin of mice after injection of Dox-HGNPs; and (**D**) Ultravist 300. Reproduced with permission from [51]. Copyright Journal of Controlled Release, Elsevier, 2015.

Recently, gold nanoparticles have been extensively used in multifunctional platforms, such as combination therapies and theranostic applications. One such combination therapy, reported by Ming *et al.* [49], used a gold NR-capped magnetic core conjugated on mesoporous silica shell exhibiting synergistic chemo- and photothermal therapy and offered combined MRI and infrared thermal imaging modalities in one system. Huiyi *et al.* [50] designed low systemic toxicity multifunctional nanocomposites comprising of gold nanoshells on silica nanorattles (GSNs). The GSNs demonstrated optical tunability and high payload with sustained drug release. Drug-loaded GSNs also showed mild low side effects and higher therapeutic effect. The triple-combination therapy, which integrates chemo-, radio-, and thermal therapy with novel metal nanoparticles, was developed by Park *et al.* [51]. A formulation of doxorubicin-loaded hollow gold nanoparticles (Dox-HGNPs) demonstrated the synergy of heat, drug, and radiation therapies. The release of Dox was triggered by an NIR laser and increased with irradiation. The radiosensitization resulted in a high level of γ-H2AX (phosphorylated histone) foci than before the irradiation, proving the radioenhancing effect of Dox-HGNPs. CT imaging studies were performed to compare the clinically available Ultravist 300 and HGNPs and concluded that Dox-HGNPs exhibited a linear dependence of the absorption on the concentration and an attenuation coefficient higher than that of Ultravist 300, as shown in Figure 2.

PTT and photodynamic therapy (PDT) are two photon-mediated therapeutic methods that can be combined in one platform for efficient cancer-killing efficiency. The integrated PTT and PDT nanoplatform aims to achieve reactive oxygen species (ROS) and hyperthermia-mediated cellular damage [52]. Table 1 demonstrates the theranostic potential of gold nanoparticles.

Although gold nanoparticles does not exhibit inherent toxicity, capping agents such as cationic ligands elicited toxicity in *in vitro* applications [71]. Therefore, a precise design considering factors such as toxicity and systemic interactions would greatly enhance the therapeutic efficacy of gold nanoparticles. Because a great variety of targeting and recognition units can be conjugated on the surface of gold nanoparticles, issues such as systemic toxicity and immunogenicity can be avoided.

### 2.2. Silver Nanoparticles

Similarly to gold nanoparticles, silver nanoparticles have photo-thermal conversion properties. Many studies have combined these two noble metals into core-shell nanostructures. Gold is often selected as the shell over a silver core because of its better NIR absorption. Galvanic repulsion and seed-mediated growth are popular techniques employed to fabricate Au/Ag core/shell structures [72,73]. Recently, Shi *et al.* [74] designed Au@Ag/Au nanoparticles for image-guided thermotherapy. Au@Ag/Au nanoparticles are formed by coating of Au NR with Ag and coated again with the Au nanolayer to increase the biocompatibility. Activatable aptamer probes containing thiolated aptamer and fluorophore-labeled cDNA were self-assembled on Au@Ag/Au, whereas the nanoparticles acted both as fluorescence quenchers and heaters. Fluorescence signal activation occurs during target recognition and thus offers on-demand PTT therapy using image-guided irradiation. In another work of Boca *et al.*, chitosan-coated triangular silver nanoparticles were synthesized; these were proven by *in vitro* results to be effective phototherapeutic agents with strong NIR resonances and exhibited an enhanced hyperthermia effect compared to PEG-capped gold NRs [75].

### 2.3. Platinum Nanoparticles

Platinum-based drugs have been used extensively in chemotherapy and, in recent times, they have drawn interest as a fluorophore and PTT of tumors. Although platinum nanoparticles exhibit antioxidative and DNA-strand breaking capacity owing to their potential toxicity, their use is not encouraged for anticancer therapy because of side effects and dose-limited toxicity [76]. By carefully controlling their size and shape, the systemic toxicity can be reduced [77,78]. Manikandan *et al.* [79] synthesized non-toxic platinum nanoparticles by a nucleation–reduction reaction of the Pt precursor and the particles showed effective photothermal killing of cells. Cancer cells reduced the platinum metal {Pt (IV)} salts to metallic nanoclusters although the mechanism of this effect is still unknown. Chen *et al.* designed a rapid one-step synthesis of fluorescent nanoclusters of platinum by the collaborative reduction of glutathione and ascorbic acid with chloroplatinic acid as a precursor [80]. They have also reported the spontaneous synthesis of biocompatible platinum nanoclusters by cancerous cells, which can be helpful in PTT and imaging. The biosynthesized nanoclusters proved to be a novel platform for image-guided PTT when combined with the porphyrin derivative tetrakis (sulfonatophenyl) porphyrin (TSPP) [81].

### 2.4. Palladium Nanoparticles

Palladium has a higher melting point and photothermal stability and has shown tunable localized SPR in the NIR region. Palladium nanosheets have been observed to be more stable than gold nanorods upon irradiation and to retain the SPR in the NIR region [82]. The lithography technique has enabled the fabrication of Pd nanodisks with tunable SPR properties. Ultrathin Pd nanosheets demonstrated SPR absorption properties [83]. By coating Pd nanosheets with Ag, their photothermal stability can be enhanced to a great extent [84]. Pd nanosheets covered by mesoporous silica nanoparticles exhibited enhanced cellular internalization and were utilized for chemo-PTT [85]. Furthermore, the structure of Pd determines its photothermal effects. Xiao *et al.* evaluated the differences of the photothermal effect of Pd nanocubes and Pd porous structures [86]. The porous Pd nanoparticles showed a two-fold enhancement in NIR absorbance than the nanocubes structure, broadband NIR absorption, and efficient photothermal conversion. Ultrasmall Pd nanosheet surfaces functionalized with reduced glutathione demonstrated prolonged blood circulation, efficient PTT in the NIR region, high accumulation in tumors, and high renal clearance [87].

### 2.5. Metal Chalcogenides

Metal chalcogenides recently received extensive attention for their role in PTT because they present excellent optical, mechanical, and chemical properties similar to those of graphene [88]. Localized SPR has been observed in chalcogenide semiconductors doped with a high concentration of free carriers [89,90]. The SPR of chalcogenide elements has long been employed in sensor applications [91]. Although metallic nanoparticles exhibit excellent photothermal properties, their biocompatibility and biological fate have been a great concern [92]. Stanley *et al.* [93] investigated the NIR photothermal properties of chemically exfoliated molybdenum disulfide (MoS_2_), which has high loading capacity, on par with graphene, owing to its high ratio of surface area to mass. PEG-functionalized MoS_2_/Fe_3_O_4_ composites (MSIO) were prepared for PTT guided by MR and PAI. The MSIOs effectively ablated the tumor upon NIR laser irradiation and showed the potential for use in MR/PA imaging [94]. PTT-triggered drug release using single-layer MoS_2_ nanosheets was reported by Yin *et al.* [95]. Chitosan-functionalized MoS_2_ sheets exhibited effective loading and controlled drug release upon NIR irradiation. Enhanced contrast was also observed in X-ray CT owing to the X-ray absorption ability of MoS_2_.

MoS_2_, which exhibits high NIR absorbance, and bismuth, which is extensively used in X-ray CT, were integrated into a theranostic system for image-guided therapy.MoS_2_/Bi_2_S_3_-PEG (MBP) composite nanosheets were synthesized with the solvothermal method and showed excellent stability and compatibility. PEGylated MBP sheets showed excellent radiosensitization and X-ray attenuation properties with good photothermal performance [96]. Oxygen-deficient molybdenum oxide (MoO_3−*x*_) has exhibited an effective localized SPR in the NIR region, which was applied in PTT for cancer [97]. PEG-functionalized MoO_3−*x*_ hollow nanospheres (PEG-MoO_3−*x*_-HNS) with intrinsic mesoporous characteristics ablated tumors efficiently and were used for showed PAI-guided chemo PTT using a camptothecin drug on pancreatic cancer [98].

**Figure 3:**
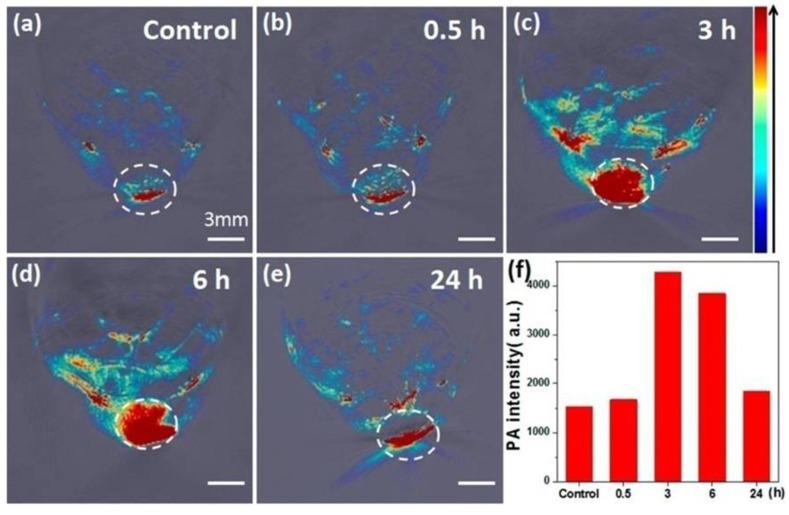
*In vivo* multispectral optoacoustic tomography (MSOT) imaging. (**a**–**e**) MSOT images of tumor before and after intravenous injection with Bi_2_S_3_ nanorods (NRs); and (**f**) photoacoustic signal intensity in tumor at different time points. Reproduced with permission from [105]. Copyright American Chemical Society, 2015.

Bismuth selenide (Bi_2_Se_3_) has long been studied in the biomedical field of biological tolerance [99,100]. Bi_2_Se_3_ nanoplates exhibit effective NIR absorption and strong X-ray attenuation. They have been utilized in X-ray CT imaging of tumor tissue [101]. Multispectral optoacoustic tomography (MSOT) is an imaging modality based on acoustic waves induced by NIR absorption and offers precision diagnosis and real-time monitoring [102,103]. Bismuth sulfide (Bi_2_S_3_) NRs were employed for bimodal imaging of MSOT and CT because of their X-ray attenuation and high NIR absorbance [104]. Tween-functionalized Bi_2_S_3_ NRs exhibited MSOT contrast and also enhanced the contrast in angiography and organic imaging *in vivo*, as shown in Figure 3 [105].

Tungsten has strong X-ray attenuation properties and high drug-loading capacity owing to its high surface area. PEGylated WS_2_ represents a new class of PTT materials with bimodal CT and PAI imaging modalities [106]. WS_2_ nanosheets were also investigated for their potential implementation in PDT and the development of a new nanomaterial for synergistic anticancer effects [107]. Tungsten oxide nanocrystals are also of great interest in NIR photoabsorption owing to their unusual defect structure [108]. PEGylated tungsten oxide nanowires (W_18_O_49_) exhibited strong NIR absorption under 980-nm laser irradiation and were used for efficient *in vivo* ablation of cancer cells [109]. WS_2_, which has a high Z number, can act both as a radiosensitizer and a PTT agent and it can be a good candidate for synergistic PTT/radiotherapy. WS_2_quantum dots (QDs) with a diameter of 3 nm efficiently improved the cancer-killing and dose-enhancement effects of radiotherapy [110].

CuS is a well-known p-type semiconductor material that has demonstrated a PTT effect under 808-nm laser irradiation [111]. The NIR absorption of CuS obtained by the d-d transition of the Cu^2+^ ions was not affected by the surrounding environment or solvent [111]. CuS nanoparticles exhibited a SPR with tunable properties that were dependent on the size and shape of the nanoparticles [112]. The first study on CuS nanoparticles for PTT was reported by Zhou *et al.* [113]. The radioactive element ^64^Cu integrated with CuS and PEG (PEG-stabilized ^64^Cu-CuS NP) demonstrated passive targeting and photothermal killing *in vitro* and *in vivo*. Table 2 elucidates the multifunctional potential of CuS nanoparticles in image-guided therapies.

## 3. Magnetic Nanoparticle-Based Thermal Therapy

Magnetic nanoparticles based thermal therapy is very well studied thermal therapy and can complement with all the available treatments such as chemotherapy, gene therapy, immunotherapy, radiation therapy, *etc.* Magnetic nanoparticles based thermal therapy possess unique advantage over conventional thermal therapies such as: (1) harmless penetration of frequencies produced by magnetic nanoparticles [120]; (2) heat generation is homogenous [121]; (3) MHT based thermal therapy may induce antitumoral immunity [122]; and (4) MHT approach helps us to develop a powerful theranostic tool by simultaneously providing thermal therapy and MRI. MHT applications need very high concentrations of Fe (around 1–2 M) [123], which is a major hurdle for human use. Recently, many investigations were carried out to minimize the concentrations of Fe for thermal therapy by formulating multicore iron oxide nanoparticles, iron oxide nanocubes, magnetic core-shell nanoparticles, *etc.* for PTT [124,125]. Crystallized form of iron oxide nanoparticles coated with polysiloxane showed an exceptional temperature rise of 33 °C with a laser power of 2.5 W/cm^2^ and exhibited enhanced PTT than commercially available magnetic nanoparticles [126]. Previously, Iron/iron oxide core-shell nanoparticles have been applied for MHT and MRI [127,128]. However, Zhou *et al.* [129] investigated the PTT efficiency of core-shell nanoparticles, which showed enhanced photothermal stability and PTT efficiency of ~20% compared to that of gold NRs. In addition, magnetic nanoparticle clusters exhibited higher NIR absorption and PTT efficiency than individual magnetic nanoparticles utilizing the fact that aggregation of metallic nanoparticles exhibits high NIR absorption for PTT [130,131].

### 3.1. Iron oxide Nanoparticles

Fe_3_O_4_ is a potential MRI candidate with high magnetic saturation and has been confirmed as a clinical magnetic contrast agent for imaging [15]. Several researchers explored the photothermal efficiency of Fe_3_O_4_ and observed that it can be used as a promising tumor treatment using NIR laser irradiation [129,131,132,133,134,135,136,137,138,139]. Additionally, a few studies demonstrated complete tumor eradication by combining chemotherapy and MHT [140].

**Figure 4:**
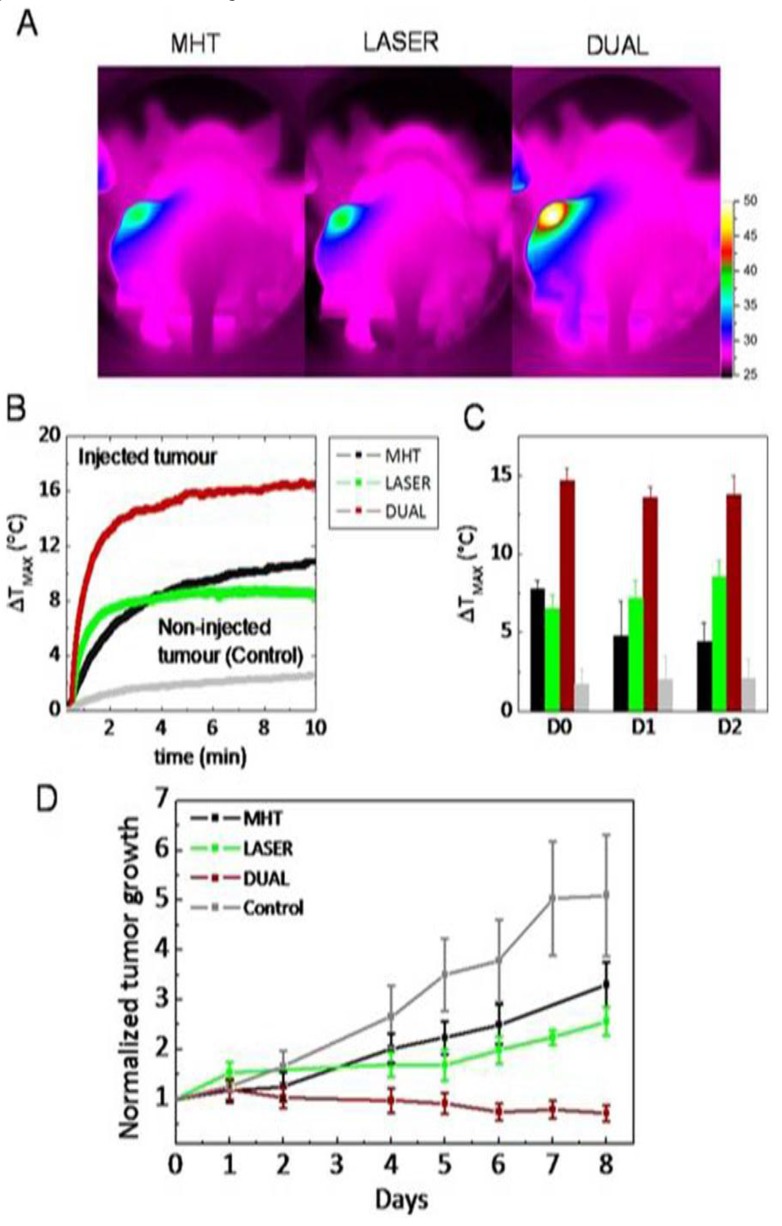
(**A**) Thermal images acquired after the intratumoral injection of nanocubes and the application of magnetic hyperthermia (MHT), near-infrared (NIR)-laser irradiation, or dual-mode treatment (both effects); (**B**) thermal elevation curves for the non-injected tumor in the dual condition; (**C**) average final temperature increase obtained on day 0 (1h after injection) and one and two days after injection for non-injected tumors; and (**D**) average tumor growth in nanocube-injected mice. Reproduced with permission from [143]. Copyright American Chemical Society, 2015.

Hayashi *et al.* [141] modified superparamagnetic iron oxide nanoparticles (SPION) clusters with folic acid and PEG (FA-PEG-SPION NCs) and generated local heating under AMF and enhanced MRI contrast with neither liver nor kidney toxicity. MHT and chemotherapy were combined with a smart nanoparticle system for synergistic therapy and achieved tumor therapy without recurrence [142]. Ana *et al.* [143] designed iron oxide nanocubes and investigated them for dual-mode treatment combining MHT and PTT. The PEG-gallol-coated nanocubes were exposed to both AMF and NIR laser irradiation and amplified the heating effect two to five times compared with MHT alone. The dual-mode treatment realized complete tumor regression mediated by apoptosis and the destruction of collagen fibers, as shown in Figure 4.

### 3.2. Magnetic Nanostructures

Recently, the importance of magnetic nanostructures in building a theranostic nanoplatform in combination with NIR absorbing materials for image-guided therapies has increased [144,145]. Photoacoustic tomography and MRI modalities have been extensively used for theranostic applications under a single platform to offer higher resolution and to depict subsurface tissue structures [146]. In a recent work by Tian *et al.* [147], multifunctional Fe_3_O_4_@Cu_2__−*x*_S core shell nanoparticles were prepared by combining PTT and MR imaging. The PTT effect can be precisely monitored and controlled by varying the Cu content in the core-shell structure. Iron carbide nanoparticles with magnetic properties were prepared by Yang *et al.* with a thin carbon shell and provided a platform for MRI,PTT, and PAI [148]. Lipid-modified iron carbide nanoparticles were produced by modifying DSPE-PEG-NH_2_ on Fe_5_C_2_ with a targeting human epidermal growth factor receptor-2 antibody (Fe_5_C_2_-ZHER2:342) for targeting ovarian cancer. The Fe_5_C_2_ probe achieved a multifunctional platform with several advantages, such as core protection from oxidation, high NIR absorption from carbon on the surface, and enhanced PTT and photoacoustic signal compared with gold nanorods. Fe_5_C_2_-ZHER2:342 exhibited improved MR contrast and efficient photothermal ablation without systemic side effects [149].

The synthesis steps of composite nanoparticles are complex and present a degradation problem. To address these issues, Yang *et al.* [150] designed magnetic iron sulfide (FeS) nanoplates with a single component and a simple one-step method for MR-imaging-guided PTT. The PEGylated FeS (FeS-PEG) nanoplates achieved high NIR absorbance and high T2 contrast compared with clinically approved contrast agents. The intravenous injection of high-dosage FeS-PEG elicited no animal toxicity and was gradually excreted through the major organs. PEG-modified iron diselenide nanoparticles (PEG-FeSe_2_) have recently emerged as potential magnetic nanostructures for dual-modal imaging and PTT. Fu *et al.* [151] synthesized FeSe_2_ by a simple solution-phase method. PEG-FeSe_2_ exhibited higher r2 relaxivity than the clinically available Feridex and showed high PAI contrast and effective PTT owing to its high NIR absorbance.

Although both photothermal and magnetic hyperthermia have demonstrated promising results, they suffer from serious drawbacks, such as high doses of laser irradiation and nanoparticle concentrations that are potentially toxic to healthy cells. Studies focusing on the reduction and optimization of iron doses with tolerable magnetic fields would achieve desired results in synergistic approaches.

## 4. NIR-Absorbing Carbon Nanomaterials for Thermal Therapy

Carbon-based nanomaterials emerged as the most promising materials for thermal therapy applications, as they impart versatile properties to the formulation such as large surface area, electrical properties and non-covalent loading of anticancer drugs. Carbon materials such as graphene was applied in *in vivo* PTT, but their applications are limited by their solubility [152]. PEGylation and polymer coating helps to attain stable dispersion and significant increase in NIR absorption [18]. Hybrid nanomaterials are designed to further enhance PTT, by integrating gold with reduced graphene oxide (rGO). This showed an increased temperature rise than nonreduced graphene oxide gold nanoparticle or noncoated graphene oxide nanoparticles [153]. Carbon nanomaterials such as carbon nanotubes (CNTs) are specially equipped with huge surface areas, which can be exploited for drug delivery applications [154]. Combined photothermal and chemotherapy appeared to exert synergistic effect on application of nanocomposite comprising of doxorubicin loaded mesoporous silica coated on single-wall carbon nanotubes (SWNTs) [155]. It was found that SWNTs in combination with anti-CTLA-4 antibody can act as immunological adjuvant and release tumor associated antigens, which can drive complete tumor cell destruction with minimum dosage of SWNTs (0.33 mg/kg) [156].

### 4.1. Graphene

Graphene nanoparticles are major conventional carbon nanomaterials used in biomedicine and imaging applications owing to their electronic properties, non-toxic *in vitro* environment, and cancer-specific drug delivery [157]. A comparative PTT study between graphene nanoparticles and CNTs was performed by Zoran *et al*. [158]. Graphene nanoparticles demonstrated superior photothermal death and efficiency due to the oxidative stress and membrane depolarization of mitochondria. In addition, reduced nanosized graphene oxide (GO) exhibited six times higher NIR absorption than non-reduced graphene and achieved PTT with lower doses [18]. One of the recently developed forms of rGO nanomesh showed an ultra-efficient PTT effect and exhibited high-fold NIR absorption compared with PEGylated rGO nanoparticles and graphene oxide [159].

A theranostic platform designed by Lin *et al.* [160] presented dual PTT properties after combining the photothermal conversion efficiencies of graphene and gold nanoparticles with PAI. Furthermore, rGO-coated gold super-particles, prepared using GO as the emulsifying agent, showed enhanced PTT properties and highly sensitive photoacoustic detection and ablation of tumors. Similarly, a smart theranostic probe based on GO and gold for fluorescent/photoacoustic image-guided PTT was synthesized by ligating gold nanoparticles on a graphene oxide surface [161]. A NIR-dye-labeled matrix metalloproteinase-14 (MMP 14) substrate was conjugated with a GO/Au hybrid to provide real-time imaging by cleaving MMP 14 and exhibited strong fluorescence in the tumor environment. Table 3 depicts the theranostic strategies designed for multimodal imaging and therapy using graphene oxide.

### 4.2. CNTs

Multi-walled carbon nanotubes (MWNTs) are cylindrical nested structures of graphene with a strong absorbance in the NIR region and have been extensively studied as a photothermal agent [178]. MWNTs have more electrons per particle on the surface than SWNTs and, hence, exhibit enhanced NIR absorption and photothermal conversion efficiency [179]. Many strategies, such as surface functionalization and coating, have been examined for the reduction of the inherent toxicity of CNTs. Fisher *et al.* proved that pluronic-coated MWNTs were effective in thermal therapy and also reduced the toxicity of CNTs [178].

PEGylated nanostructures reduce systemic toxicity and provide efficient therapy. PEGylated MWNTs were designed by Zhen *et al.* [180] for the photothermal ablation of bone metastasis in breast cancer. They observed enhanced suppression of tumor growth and low systemic toxicity compared to bare MWNTs. MWNTs for a lymphatic theranostic system were developed by Sheng *et al.* by coating MWNTs with manganese oxide and PEG; the authors reported simultaneous imaging by T1-weighted MR imaging of MnO and dark-dye imaging of the MWNTs with NIR ablation through dual-modality mapping [181]. A theranostic nanoplatform based on magnetic MWNTs was demonstrated by Lei *et al*. [182]. Magnetic nanoparticles conjugated with the MWNT surface were modified with PEI and PEG to attain biocompatibility. The human telomerase reverse transcriptase small interfering RNA (siRNA)-loaded MWNTs achieved efficient delivery of the siRNA along with PTT heating and MR imaging.

A few studies have reported on the PTT efficacy of SWNTs despite the fact that their light-to-heat conversion efficiencies are lower than those of MWNTs because of the superior electrical properties of MWNTs. Chao *et al.* [183] studied metastatic sentinel lymph nodes thermally ablated using SWNTs, which showed enhanced retention, MR contrast, and pulmonary metastasis inhibition. Similarly, Antaris *et al.* [184] used (6,5) chirality SWNTs after modification with poly(maleic anhydride-alt-1-octadecene)-methoxy PEG (C18-PMH-mPEG) surfactant to generate biocompatible SWNTs. The chirality-sorted CNTs exhibited bright fluorescence and ablation temperature with an injected dose more than ten times lower than that of synthesized SWCNTs.

Owing to their large surface area, large electrical conductivity, and high drug loading, carbon nanomaterials have been proven to be very efficient for combination and multifunctional therapies. However, carbon-based nanomaterials present potential toxicity and bioavailabilty issues. Surface coatings of appropriate biocompatible and biological molecules can reduce the toxicity and be excreted over time.

## 5. QDs-Based Thermal Therapy

QDs were primarily developed as fluorescent probes. They have been used as probes for photothermal and photoacoustic contrast agents and sensitizers and provide multimodal therapy and a diagnostic platform [185]. QDs are resistant against photobleaching and their narrow emission spectra are beneficial in photo-based treatments owing to their size-dependent and strong fluorescent properties [186]. Chu *et al.* [187] studied the photothermal potential of CdTe and CdSe QDs and evaluated their therapeutic efficiency in melanoma. After laser irradiation, a temperature increase and intracellular ROS production were generated together with tumor inhibition. Sun *et al.* [188] recently demonstrated the photothermal potential of black phosphorous QDs and their appreciable photothermal conversion efficiency. Transition metal dichalcogenides have been investigated as PTT agents and some have demonstrated to be excellent candidates for radiosensitization [95,107]. Yuan *et al.* developed multifunctional tungsten sulfide QDs (WS_2_ QDs) for dual-mode imaging and synergistic therapy combining PTT and radiotherapy. WS_2_ QDs exhibited a signal enhancement in X-ray CT/PAI. Intravenous injections of QDS eradicated the tumor and facilitated the multimodal imaging and synergistic therapy [110].

**Figure 5:**
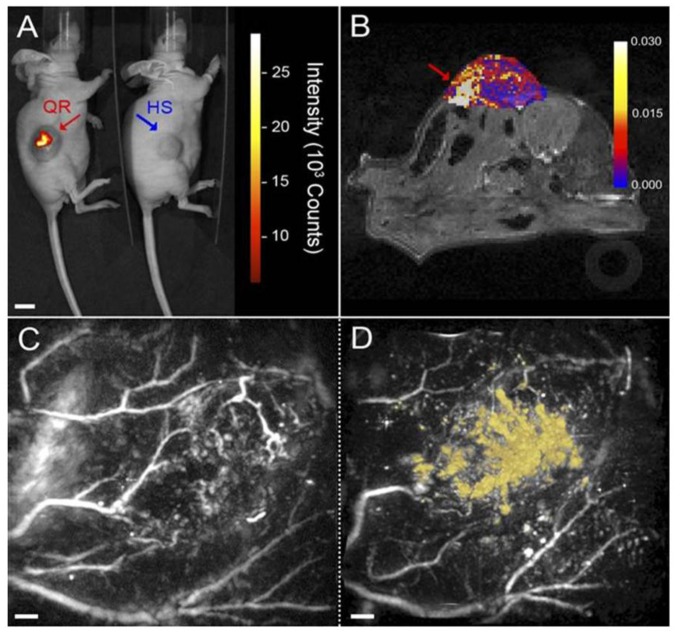
Multimodal *in vivo* imaging of quantum rattles (QRs): (**A**) NIR fluorescent intensity in the areas where QRs (red) and non-fluorescent hollow mesoporous silica shells (HS) control (blue); (**B**) MR image obtained following the injection of QRs; and 3D photoacoustic images of tumors acquired at 670 nm before (**C**) and after (**D**) the injection of QRs. Reproduced with permission from [191]. Copyright Proceedings of the National Academy of Sciences of the United States of America, 2015.

Gold QDs have enhanced optical and magnetic properties compared to the larger gold nanoparticles although the applications of Au QDs are limited by their aggregation and unfavorable interactions in aqueous solvents [189,190]. Mathew *et al.* [191] designed a gold-silica rattle (quantum rattle, QR) consisting of a hollow mesoporous silica shell with two hydrophobic domains of Au QDs and larger gold nanoparticles to retain the advantages of Au QDs. Furthermore, the drug-carrying efficiency and prolonged release of the drug were achieved because of the highly hydrophobic surface of the QR. Figure 5 shows the multimodal *in vivo* imaging of QRs in a colorectal carcinoma tumor model. The NIR fluorescence and photoacoustic images clearly demonstrate strong post-treatment contrast of the QRs within the tumor mass compared to the hollow silica shell-treated animals.

## 6. UCNPs-Based Thermal Therapy

UCNPs emit short-wavelength photons upon NIR light excitation and thus provide a new scope in biomedical imaging [192]. UCNPs have several advantages compared to organic dyes, such as narrow emission peaks, good photostability, high signal-to-noise ratio, and low toxicity [193]. The emission and therapeutic efficiency of UCNPs can be enhanced by surface coating with gold nanoparticles and the loading of drugs or photosensitizers on UCNPs for multimodal imaging and therapy [194,195]. Multifunctional nanoparticles (MFNPs) have been formed using layer-by-layer assembly of UCNPs as the core, ultrasmall iron oxide nanoparticles as the intermediate shell, and Au as the outer shell. The MFNPs exhibited UCL, MR, and photothermal ablation of tumor cells [196]. Additionally, *in vivo* multimodal imaging and efficient PTT were achieved using MFNPs, which showed no systemic toxicity [197].

Theranostic UCNPs were prepared by Yinghui *et al.* by covalently grafting nanographene oxide (NGO) to core-shell UCNP and loading phthalocyanine (ZnPc) on NGO. The UCNPs-NGO/ZnPc were used as UCL probes, and resulting PDT and PTT showed high therapeutic efficiency in *in vitro* cancer therapy [198]. Protein-modified UCNPs (NaGdF_4_:Yb:Er) were also employed for synergistic PTT and PDT by simultaneously loading the photosensitizer rose bengal and NIR dye IR825 [199]. Multifunctional nanostructures based on DNA backbones were designed for multimodal image-guided therapy. A core-satellite structure, in which the core was composed of gold NRs and chlorine e6-attached UCNPs (NaGdF_4_) as satellites, was assembled hierarchically by complementary base pairing. Combined with UCL, MRI, CT, and PAI, the core-satellite structures achieved the complete elimination of tumors with a safe dosage [200].

Organic-inorganic nanocomposites based on UCNPs were prepared for synergistic therapy by Liu *et al.* [201]. The nanocomposites, formed by doxorubicin-loaded NaGdF_4_:Yb, Er@NaGdF_4_UCNP@PDA core-shell nanoparticles (UCNP@PDA5-PEG-DOX), were suitable five applications: UCL, MRI, CT, PTT, and chemotherapy. The nanocomposites elicited no organ toxicity and enhanced the tumor cytotoxicity without regrowth. Photothermal therapy and radiotherapy were integrated under one platform by decorating CuS nanoparticles on silica-coated rare earth UCNPs (NaYbF_4_:2%Er^3+^/20%Gd^3+^@SiO_2_-NH_2_). The synergistic interaction between radiotherapy and PTT eradicated tumors in mice with negligible toxicity by simultaneously providing UCL, MRI, and CT [202].

## 7. Conclusions and Perspectives

An overview of the different classes of inorganic nanoparticles used in thermal therapy and imaging has been presented. Considering the great variety of nanoparticles and parameters used for thermal therapy, it is difficult to focus on a single particle for an improved therapy. In the future, PTT can be combined with immunotherapy using immunoadjuvants with PTT agents to produce a synergistic anti-tumor effect. Despite exhibiting a tremendous potential in thermal-induced therapies, research on inorganic nanoparticles must address many issues, such as photostability, physiological stability, and clearance, before proceeding into clinical trials. The stability of inorganic nanoparticles is a potential advantage over conventional ones although their long-term toxic effects must be investigated. Although a few inorganic nanoparticles are in clinical use, such as iron oxide in MRI, clinically relevant issues, such as systemic toxicity and clearance, must be addressed before promoting thermal therapies for clinical use. To sum up, inorganic nanoparticles have demonstrated tremendous potential as a theranostic tool and have revealed a new direction in cancer therapy.

## Figures and Tables

**Table 1:** Various types of multifunctional gold nanoparticles used in image-guided therapies.

Nanomaterials	Therapy	Imaging modality	Ref.
Ce6-loaded gold vesicles (GV-Ce6)	PTT/PDT	Fluorescence/thermal/PAI	[53]
Ce6 conjugated aptamer functionalized gold NR	PTT/PDT	Fluorescence imaging	[54]
Gold NR-photosensitizer complex (GNR-AIPcS4)	PTT/PDT	Fluorescence imaging	[55]
Chitosan functionalized pluronic nanogel-loaded gold NRs and Ce6	PTT/PDT	Thermal/fluorescence imaging	[56]
Gold nanoshelled microcapsules	PTT	Thermal/ultrasound imaging (USI)	[57]
Cyclic RGD conjugated gold nanostar (RGD-GNS)	PTT	Thermal/PAI	[58]
Gold NRs and conjugated poly(styrene-alt-maleic acid) and ICG	PTT	Two-photon luminescence	[59]
CD44v6-conjugated PEG-modified gold nanostars	PTT	PAI/ Infrared microscopic imaging	[60]
Gold NR-encapsulated protein-shell microbubbles	PTT	PAI/two-photon fluorescence	[61]
Gold-poly dopa core-petal nanostructures	PTT/PDT	Fluorescence imaging	[62]
Gold nanostars	PTT/PDT	X-ray imaging/fluorescence imaging	[63]
Methylene blue-loaded gold NR-SiO_2_ core-shell nanocomposites	PTT/PDT	Fluorescence imaging	[64]
(MB-GNR@SiO_2_)			
Chlorin e6 conjugated gold nanostars (GNS-PEG-Ce6)	PTT/PDT	Fluorescence imaging/US imaging/PAI	[65]
Super paramagnetic Fe_3_O_4_ welding on Au shells with polyphosphazene as coating agent	PTT	MRI	[66]
Gold colloids coated on polystyrene sphere modified with chitosan and containing Fe_3_O_4_	PTT	MRI/dark field imaging	[67]
Hyaluronic acid-modified Fe_3_O_4_—Au core/shell nanostars	PTT	MRI/CT/thermal imaging	[68]
Core-shell Fe_3_O_4_—mSiO_2_ nanoparticles	PTT	MRI	[69]
Core-shell structure	PTT	MRI/CT	[70]
Core: Gold nanoparticles coated with polydopamine			
Shell: ICG and functionalized lipids with gadolinium and lactobionic acid			

**Table 2:** Therapeutic and imaging potential of CuS nanoparticles.

Nanomaterials	Therapy	Imaging Modality	Ref.
Copper sulfide nanodot (CuS)	PTT	Positron emission tomography (PET)	[114]
Folic acid onto the surface of mesoporous silica-coated core-shell-shell upconversion nanoparticles (UCNPs) with Dox loading	PTT/chemo therapy	Up-conversion luminescence (UCL), CT, and MRI	[115]
Chelator-free multifunctional (^64^Cu) CuS nanoparticles	PTT	Micro-PET/CT	[113]
Ultrasmall Cu_(2−*x*)_S nanodots (u-Cu_(2−*x*)_S)	PTT	PAI	[116]
Dox-loaded Cu_9_S_5_@mSiO_2_@Fe_3_O_4_-PEG	PTT/chemo therapy	MRI	[117]
PEGylated CuS nanoparticles	PTT	PAI	[118]
Ultrasound-targeted microbubbles depositing CuS nanoparticles	PTT	USI	[119]

**Table 3:** Graphene nanoparticles in cancer theranostics.

Nanomaterials	Therapy	Imaging modality	Ref.
rGO-loaded ultra small plasmonic gold NR vesicle	PTT	Ultrasound/photoacoustic	[162]
Graphene oxide/manganese ferrite nanohybrids	PTT/drug	MRI	[163]
Iodine-labelled rGO	PTT/radiotherapy	Gamma imaging	[164]
Indocyanine green loaded onto hyaluronic acid-anchored rGO(HArGO) nanosheets (ICG/HArGO)	PTT	Fluorescence imaging	[165]
2-chloro-3-4-dihydroxyacetophenone quaternized poly(ethylene glycol)-grafted poly(DMAEMA-co-NIPAAm) (CPPDN)-complexed Indocyanine green (ICG-CPPDN/rGO)	PTT	Fluorescence imaging	[166]
Nano-graphene oxide—Tf-FITC	PTT	Fluorescence imaging	[167]
rGO-coated gold NRs	PTT	PAI	[168]
Graphene oxide—BaGdF_5_ nanocomposites	PTT	MRI/ X-ray CT imaging	[169]
Graphene oxide modified with iron oxide nanoparticles (GO-IONP)	PTT	MRI	[170]
Carboxylated photoluminescent graphene nanodots	PTT/PDT	Photoluminescence	[171]
Tris(2,2′-bipyridyl)ruthenium-(II)chloride (Rubpy)/GO nanohybrid	PTT	Surface-enhanced Raman scattering imaging	[172]
IL-13 peptide-modified magnetic graphene-based mesoporous silica (MGMSPI)	PTT/drug	MRI	[173]
BSA-functionalized nano-rGO	PTT	PAI	[174]
Graphene oxide—iron oxide nanoparticle-gold nanocomposite (GO-IONP-Au)	PTT	MRI/X-ray imaging	[175]
Graphene-oxide-modified PLA microcapsules	PTT	Ultrasonic/CT Imaging	[176]
rGO—iron oxide nanoparticle (IONP) nanocomposite non-covalently functionalized with PEG (RGO–IONP–PEG)	PTT	MRI/PAI	[177]

## Abbreviations

The following abbreviations are used in this manuscript:

US	Ultrasound
MR	magnetic resonance
CT	computed tomography
QD	quantum dot
UCNP	upconversion nanoparticles
CuS	copper sulfide
CNT	carbon nanotube
AMF	alternate magnetic field
ROS	reactive oxygen species
SPR	surface plasmon resonance
NIR	near-infrared
PAI	photoacoustic imaging
MRI	magnetic resonance imaging
PTT	photothermal therapy
DAMPs	damage-associated molecular pattern molecules
rGO	reduced graphene oxide
NGP	Nanoscale gold particles
GSNs	gold nanoshells on silica nanorattles
PDT	photodynamic therapy
USI	ultrasound imaging
MSOT	Multispectral optoacoustic tomography
PET	Positron emission tomography
UCL	Up-conversion luminescence
SPION	superparamagnetic iron oxide nanoparticles
MMP 14	matrix metalloproteinase-14
MWNTs	Multi-walled carbon nanotubes
SWNTs	single-wall carbon nanotubes
siRNA	small interfering RNA
QR	quantum rattle
MFNPs	Multifunctional nanoparticles
